# Preserving effect of Loboob (a traditional multi-herbal formulation) on sperm parameters of male rats with busulfan-induced subfertility

**DOI:** 10.5935/1518-0557.20210099

**Published:** 2022

**Authors:** Soghra Bahmanpour, Mojtaba Keshavarz, Farhad Koohpeyma, Parmis Badr, Adel Noori, Mohammad Hossein Dabbaghmanesh, Tahereh Poordast, Fateme Sadat Najib, Najaf Zare, Niloofar Namazi, Bahia Namavar Jahromi

**Affiliations:** 1 Anatomy Department, School of Medicine, Shiraz University of Medical Sciences, Shiraz, Iran; 2 Infertility Research Center, Shiraz University of Medical Sciences, Shiraz, Iran; 3 Endocrine and Metabolism Research Center, Shiraz University of Medical Sciences, Shiraz, Iran; 4 Pharmaceutical Sciences Research Center, Shiraz University of Medical Sciences, Shiraz, Iran; 5 Phytopharmaceutical Technology and Traditional Medicine Incubator, Shiraz University of Medical Sciences, Shiraz, Iran; 6 Student Research Center, Shiraz University of Medical Sciences, Shiraz, Iran; 7 Department of Obstetrics and Gynecology, School of Medicine, Shiraz University of Medical Sciences, Shiraz, Iran; 8 Department of Biostatistics, School of Medicine, Shiraz University of Medical Sciences, Shiraz, Iran

**Keywords:** infertility, Loboob, male subfertility, sperm, traditional Persian medicine

## Abstract

**Objective:**

Male infertility secondary to exposure to gonadotoxic agents during reproductive age is a concerning issue. The aim of this experimental study was to determine the effect of Loboob on sperm parameters.

**Methods:**

55 healthy rats were selected, weighted and divided into five groups consisting of 11 rats each. The control group received no medication. Rats in Treatment Group 1 received 10mg/kg Busulfan and rats in Treatment Groups 2, 3, and 4 received 35,70 and 140 mg/kg Loboob respectively in addition to 10mg/kg Busulfan. Finally, the sperm parameters and weights of the rats were compared using the Kolmogorov-Smirnov, non-parametric Kruskal-Wallis, and Dunn-Bonferroni tests.

**Results:**

All sperm parameters and weights were significantly decreased among rats receiving Busulfan. All dosages of Loboob were effective to enhance the motility of slow spermatozoa, while only in the rats given 70 and 140 mg/kg of Loboob saw improvements in progressively motile sperm percentages (0.024 and 0.01, respectively). Loboob at a dosage of 140mg/kg improved sperm viability. It did not improve normal morphology sperm or decrease immotile sperm counts. Loboob did not affect mean rat weight.

**Conclusions:**

Loboob offered a dose-dependent protective effect on several sperm parameters in rats with busulfan-induced subfertility.

## INTRODUCTION

Infertility is an important health problem with a prevalence of 10-15% worldwide (Tansaz *et al*., 2016). Cancer may lead to infertility through alterations to the proteomic profile of spermatozoa or gonadotoxic agents used in cancer treatment. Moreover, the cancer type and behavior, the kind of cytotoxic agents and dosages used, along with patient age and general health status may affect the intensity of induced reproductive conditions. In recent years, noticeable improvements in cancer management have led to longer survival, and fertility preservation has gained renewed relevance among initiatives devised to improve the quality of life of survivors (Martins *et al*., 2020; Ozdemir & Bozdag, 2020).

A multi-herbal formulation locally called Loboob, a preparation containing specific proportions of different parts of eighteen medicinal plants processed with sugar and honey (Talib *et al*., 2017) to make a 20-ingredient semisolid compound (Bashir & Afrin, 2019; Fahamiya *et al*., 2016), has been used in Traditional Persian Medicine (TPM) to treat male infertility (Rahman *et al*., 2014). Loboob has other spellings, such as Laboob or labub. There are official formulations of Loboob in the traditional medicine market, specifically in India and Iran. Although we found no articles published in English about the ingredients of Loboob, several studies on traditional medicine in Asia contain references to Laboob-e-kabeer and Laboob-e-Sagheer (Akhtar *et al*., 2010; Bano *et al*., 2018; Bashir & Afrin, 2019; Fahamiya *et al*., 2016; Rahman *et al*., 2014; Talib *et al*., 2017). The formulation of Laboob-e-kabeer includes 60 ingredients, while Laboob-e-Sagheer is made from 20 ingredients. The Loboob formulation used in our study is a modified form of Laboob-e-Sagheer as explained in the *Qarabadin Salehi* with minor modifications (Zargaran & Zarshenas, 2017).

To the best of our knowledge, no report has been previously published on the effect of Loboob on chemotherapy-induced male infertility (Bioos *et al*., 2015; Daneshfard & Jaladat, 2016). This study aimed to analyze the potential preservative power of Loboob on the sperm parameters of rats treated with a gonadotoxic alkylating agent.

## MATERIALS AND METHODS

### Animals

This study included 55 male Sprague-Dawley rats with an average weight of 225±25 gr at the animal laboratory of Shiraz University of Medical Sciences (SUMS). The study was conducted in accordance with the National Institutes of Health guidelines (Ethics Committee certificate of approval: IR.SUMS.REC.1393.7468). The rats were kept for two weeks in the animal laboratory until they adjusted to new conditions. During this period, the experiment rats were kept under standard humidity conditions in a 12-h light and darkness cycle, housed in 20×30×55 cm Makrolon cages with stainless steel lattice roofs and wire mesh floors. The cages were disinfected with washing solution every other day. They were kept at a temperature of about 25 degrees centigrade with free access to food (Daniran feeds and foods, Shiraz, Iran) and filtered water.

### Herbal Extract

The ingredients of the product were purchased from a herbal market in Shiraz, Iran. After identification (see voucher numbers in Table 1), plant specimens were deposited at the herbarium of the Phytopharmaceutical and Traditional Medicine incubator of SUMS. The scientific names of the plants were checked on http://www.theplantlist.org. The ingredients of Loboob were prepared based on a compound named Laboob-e-sagheer described in a medieval Persian pharmacopeia (*Qarabadin Salehi*) with minor modifications and removal of potentially harmful ingredients (Zargaran & Zarshenas, 2017). Table 1 shows the scientific and English names of the ingredients, the used plant parts, and their proportions in the final product.

**Table 1.** Loboob Ingredients and their families, local name, used parts, percentages, main constituents and voucher numbers. Proportion of each ingredient showed in percentage.

	Ingredient	Family	Local name	Used part^#^	Main constituents	Percentage	Voucher No.
1	*Allium cepa* L.	Amaryllidaceae	onion	s	fatty acids, phenolic compounds (Yalcin & Kavuncuoglu, 2014)	3.45	TMI 10P7
2	*Alpinia officinarum* Hance	Zingiberaceae	lesser galangal	r	Volatile oil, phenolic compounds (Abubakar *et al*., 2018)	3.45	PTMI 108
3	*Boswellia carterii *Birdw.	Burseraceae	frankincense	o	Volatile oil, boswellic acids (Iram *et al*., 2017)	3.45	PTMI 109
4	*Brassica rapa* L.	Brassicaceae	turnip	s	Fatty acids (Basnet *et al*., 2016)	6.90	PTMI 118
5	*Cinnamomum zeylanicum* Blume	Lauraceae	cinnamon	b	Volatile oil (Ribeiro *et al*., 2020)	3.45	PTMI 110
6	*Cocos nucifera* L.	Arecaceae	coconut	f	Fatty acids (Roopan, 2016)	6.90	PTMI 101
7	*Corylus avellana* L.	Betulaceae	hazelnut	k	Fatty acids (Granata *et al*., 2017)	6.90	PTMI 102
8	*Juglans regia* L.	Juglandaceae	walnut	k	Fatty acids (Al-Snafi, 2018)	6.90	PTMI 103
9	*Lepidium perfoliatum* L.	Brassicaceae	clasping pepperweed	s	Fatty acids, gum (Lazzeri *et al.*, 2013)	6.90	PTMI 111
10	*Mentha piperita* L.	Lamiaceae	mint	l	Volatile oil (Mimica-Dukić et al., 2003)	6.90	PTMI 112
11	*Piper cubeba* L.F.	Piperaceae	tailed pepper	f	Phenolic compounds, alkaloid, lignans (Nahak & Sahu, 2011; Elfahmi *et al.*, 2007)	3.45	PTMI 113
12	*Piper nigrum* L.	Piperaceae	pepper	f	Volatile oil, alkaloid (Meghwal & Goswami, 2013)	3.45	PTMI 114
13	*Pistacia vera* L.	Anacardiaceae	pistachio	k	Fatty acids (Shokraii, 1977)	6.90	PTMI 104
14	*Prunus dulcis* Mill.	Rosaceae	almond	k	Fatty acids (Mexis *et al*., 2011)	6.90	PTMI 105
15	*Sesamun indicum* L.	Pedaliaceae	sesame	s	Fatty acids (Uzun *et al.*, 2007)	6.90	PTMI 106
16	Sugar	-	-------	-	----------------------	6.90	-
17	*Syzygium aromaticum* (L.) Merr.& L.M.Perry	Myrtaceae	cloves	bu	Volatile oil, phenolic compounds (Cortés-Rojas *et al*., 2014)	3.45	PTMI 115
18	*Withania somnifera* (L.) Dunal	Solanaceae	ginseng	r	Steroidal lactones, flavonoids, phenolic compounds (Dhanani *et al*., 2017)	3.45	PTMI 116
19	*Zingiber officinale* Roscoe	Zingiberaceae	ginger	rh	Volatile oil (Ali *et al*., 2008)	3.45	PTMI 117
20	Honey	-	--------	-	---------------------------	(× 3) of extract	-

The ingredients were washed and dried thoroughly. Each was ground to a powder separately on a grinder, and then blended together into a homogenous mixture. A hydroalcoholic (70%) extract of the powder was obtained after 72 h and submitted to a concentration process using a rotary apparatus (Büchi, Switzerland) at 37°C. The concentrate was mixed with dewaxed honey (1:3) and homogenized through continuous stirring for 30 minutes. According to TPM clinical instructions, the final product was kept at room temperature in an airtight glass container for 40 days before use.

### Inducing Subfertility With Busulfan

Similar to other alkylating agents, Busulfan may induce subfertility or infertility depending on length of exposure and dosage. According to our previous study on the toxic effects of different dosages of busulfan on the testicles of rats (Bahmanpour *et al*., 2017), we decided to use a single dose of 10 mg/kg intraperitoneal injection of busulfan in the rats included in this study to induce moderate reversible subfertility (Sigma-Aldrich Co, St. Louis, MO, USA).

### Animal Allocation

This is a prospective randomized controlled study. Calculations performed on a statistical software package considering a margin of error of 5%, statistical power of 80%, and an effect size of 10% yielded a sample size of 50 rats. Considering the possibility of sample shedding due to the prolonged nature of this study, we included 55 rats randomly assigned into five groups, each containing eleven rats, as follows:

1) Control group (C) received no medication.
2) Treatment group 1 (T1) received a single 10 mg/kg intraperitoneal injection of Busulfan.
3) Treatment group 2 (T2) received a single 10 mg/kg intraperitoneal injection of Busulfan and Loboob (35mg/kg/day).
4) Treatment group 3 (T3) received a single 10 mg/kg intraperitoneal injection of Busulfan and Loboob (70 mg/kg/day).
5) Treatment group 4 (T4) received a single 10 mg/kg intraperitoneal injection of Busulfan and Loboob (140mg/kg/day).

### Drug Administration

Busulfan was administered in a single dose via intra-peritoneal injection on the first day of the study, while Loboob was given by gastric lavage starting from the 30^th^ day of the study for a period of 60 days. The total duration of the study was 104 days, with 90 days occurring after busulfan injection. The animals were weighed before busulfan injection and at the end of the study using a compact electronic scale with an accuracy of 0.01gr (SF-400C, Suofei electronic, Jiangsu, China).

### Sperm Suspension Preparation

The rats were anesthetized with ether and slaughtered. They were placed in a supine position and had their abdomens cleaned with 70% ethanol; a vertical incision was made on the abdomen so that the fat layer and the connective tissue surrounding the vas deferens were resected. An incision was made 5cm from the epididymis following the removal of the fat layer. A petri dish containing 5 mL of Ham's F-l0 medium (Gibco BRL, Grand Island, Introduction NY, USA) was used to provide for the appropriate distribution of spermatozoa into the solution after transferring the primary 10-millimeter segment of the ductus deferens and epididymis. The spermatozoa were milked by squeezing with a forceps, which led to sperm exiting from the end of the tissue specimen. After one hour, the spermatozoa were hyperactive and distributed. The epididymis was rapidly transferred to a petri dish containing 1 ml of RPMI-1640 and 100mg/ml of BSA medium. This petri dish was placed in an incubator set at 37°C.

### Sperm Viability

Sperm viability was assessed based on eosin-nigrosin staining. One quantity of sperm suspension and two quantities of formulated 1% eosin with concentrated water (Merck, Darmstadt, Germany) were mixed. Then, an equal quantity of prepared nigrosin (Merck, Darmstadt, Germany) was poured into the mentioned mixture after 30 seconds. Qualified thin slides were inspected under a light microscope (Nikon E-200, Japan) at 40x magnification. In each sample, 100 spermatozoa were counted to determine the percentage of viable sperm. A colorless feature in spermatozoa indicated viability, while discoloration toward red indicated non-viable spermatozoa (WHO, 1999).

### Sperm Motility

The sperm suspension was transferred onto a slide, which had been previously heated to 37°C. Five microscopic fields were randomly studied to determine the motility of 200 spermatozoa in each sample using a light microscope (Nikon E-200, Japan) at 200x magnification. Classification was performed based on the moving style of sperm. Spermatozoa with rapid linear, slow linear, or circular movements were labeled as rapid progressive, slow progressive, or non-progressive, respectively. Neither linear nor circular sperm progression was marked as immotile (Momeni & Daneshpajoh, 2012).

### Sperm Count

Sperm count was performed using a Neubauer hemocytometer (Deep 0.1 mm, LABART, Germany) at 400x magnification using a light microscope. The number of spermatozoa in four squares of the Neubauer chamber were counted. The mean was multiplied by 10^6^ to obtain the number of sperm cells per mL of semen. The whole sperms (with heads and tails) were counted. Counting was done based on WHO protocol (WHO, 1999).

### Sperm Morphology

Slides of the suspension stained with 1% eosin Y were prepared and dried in room air. Then the slides were fixed with 96% ethanol and stained with the Papanicolaou method. The slides were viewed on a light microscope at 1000x magnification and the proportions of sperm with normal morphology were calculated. Sperm with unstructured or dual heads, combined bodies and dual, segmented or angled tails were considered abnormal (Momeni & Daneshpajoh, 2012).

### Statistical Analysis

At first, the normal distribution of data was evaluated with the Kolmogorov-Smirnov test. Then, the Kruskal-Wallis non-parametric test was performed to show whether there was at least one group that differed from the others, shown by a *p*-value <0.05. Finally, a post-hoc test (Dunn-Bonferroni) was performed to determine the exact effect of Loboob on each parameter and group. Statistical analysis was performed using SPSS version 22.0 and *p*<0.05 was deemed significant.

## RESULTS

Forty-nine of the 55 rats included in the study survived until final analysis. All rats included in groups C, T1 and T4 and 16 rats (eight from each group) from the T2 and T3 groups survived until final analysis.

The Kruskal-Wallis test showed that except for non-progressive motility (*p*-value=0.136), other variables had statistically significant values, which led to additional analysis with the Dunn-Bonferroni test. Loboob 70 and 140mg/kg/day were powerful enough to significantly improve the mean proportions of progressive sperm (*p*-value=0.024 and 0.01, respectively) (Table 2).

**Table 2.** Sperm parameters of rats with Busulfan-induced subfertility treated with different Loboob dosages.

Variables	Control	Busulfan	Busulfan + 35mg/kg Loboob	Busulfan + 70mg/kg Loboob	Busulfan + 140mg/kg Loboob	*p*-value^1^
Sperm Count×106 (n)	87.20±10.61	22.00±6.47▲	45.25±8.77	53.25±20.19	60.81±34.28	0.000
Progressive sperm movement (%)	36.80±19.06	1.36±3.10▲^,^●^,^*	15.25±16.88	16.12±8.57	19.45±9.75	0.000
Slow sperm movement (%)	10.42±1.98	1.09±1.64▲^,^■^,^●^,^*	8.87±4.73	9.37±5.42	11.90±10.59	0.000
Non-progressive sperm (%)	22.66±6.20	13.36±17.25	26.75±8.11	27.00±2.23	24.00±9.01	0.136
Immotile sperm (%)	6.00±2.70 Ͱ	26.87±16.60▲	24.37±11.62	17.75±7.38	10.44±8.07	0.001
Normal sperm morphology (%)	85.60±7.96 Ͱ	57.90±22.25▲	61.75±8.87	66.81±6.70	73.22±8.17	0.000
Sperm viability (%)	92.70±5.85 Ͱ^,^¥	59.83±7.13▲^,^*	68.31±12.33	71.56±7.10	78.04±6.38	0.000
Weight of rats (gr)	484.09±53.85 Ͱ^,^¥^,^#	380.66±32.16▲	382.62±23.07	388.42±27.68	391.00±39.29	0.000

1 Kruskal-Wallis test used

The values are mean±SD

▲ : Significant between control and Busulfan,

■ : Significant between Busulfan and Busulfan +35mg/kg Loboob,

● : Significant between Busulfan and Busulfan +70 mg/kg Loboob,

* : Significant between Busulfan and Busulfan +140 mg/kg Loboob,

Ͱ : Significant between control and Busulfan +35 mg/kg Loboob,

¥ : Significant between control and Busulfan +70 mg/kg Loboob,

# : Significant between control and Busulfan +140 mg/kg Loboob

All dosages of Loboob in the T2, T3 and T4 groups had statistically significant effects to improve mean slow motile sperm count (*p*-value<0.005). Loboob at a dosage of 140mg/kg/day improved sperm viability. At the dosages prescribed in this study, Loboob did not improve normal morphology or count or decrease immotile sperm count significantly (*p*-value>0.005).

**Figure 1:**
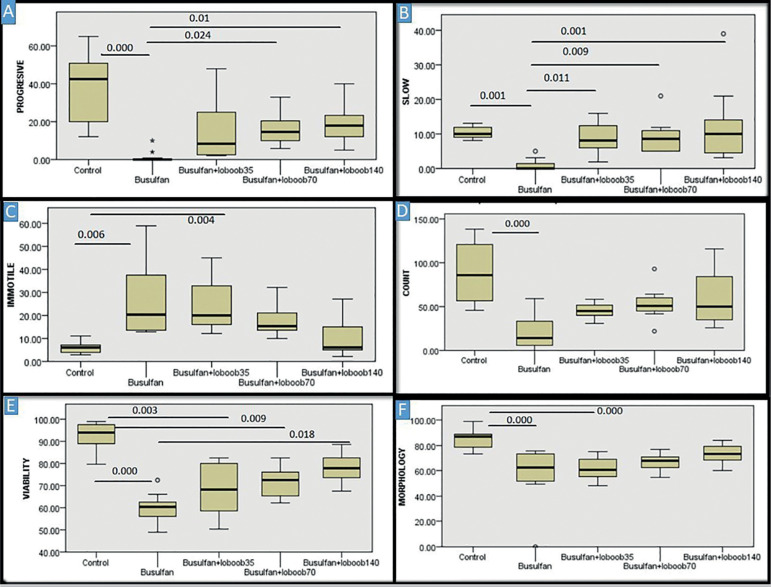
Result of post-hoc test (Dunn-Bonferroni) comparing each treated group for sperm parameters. *p*-values between the groups are shown on the line drawn to connect every compared two columns. A: *p*-values for progressive motility sperm B: p-values for slow sperm C: p-values for immotile sperm D: *p*-values for sperm count E: *p*-values for viability F: *p*-values for normal morphology.

**Figure 2:**
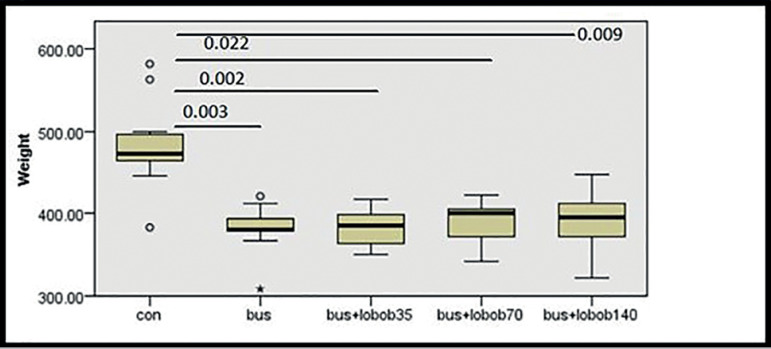
Result of post-hoc test (Dunn-Bonferroni) comparing each treated group in the aspect of body weight. *P*-values between the groups are shown on the line drawn to connect every compared two columns.

Mean body weight was not affected by Loboob in our study. The intergroup details of positive *p*-values are demonstrated in Figures 1 and 2.

## DISCUSSION

Chemotherapy-induced infertility has been mentioned as a burden on the quality of life of men of reproductive age. Some traditional medicine prescriptions are suggested to produce protective effects on semen parameters against specific chemotherapy agents (Ferdosi Khosroshahi *et al*., 2013; Mohammadghasemi *et al*., 2010; Qu *et al*., 2018; Zhang *et al*., 2020). In the present project, semen parameters were studied in rats with Busulfan-induced subfertility given Loboob at different dosages. Loboob improved the motility of slow sperm in all three used dosages in this study, but progressive sperm and viability were improved only with higher dosages. There are official formulations of Loboob in the traditional medicine market. Laboob-e-kabeer and Laboob-e-Sagheer have been mentioned in traditional books and several reports on traditional medicine in Asia (Akhtar *et al*., 2010; Bano *et al*., 2018; Bashir & Afrin, 2019; Fahamiya *et al*., 2016; Rahman *et al*., 2014; Talib *et al*., 2017) and listed under compound formulations or *Murakkabat (*Laboob-e-kabeer, Laboob-e-Sagheer) (Bashir & Afrin, 2019).

Laboob-e-kabeer as a sexual desire invigorative (Muqawwi-e-Bah) with its aphrodisiac formulation was mentioned in previous studies (Rahman *et al*., 2014). Also, in our country, Loboob is a multi-herbal compound that can be prepared with several formulations based on TPM. We used a formulation with 20 components. The scientific and common names of the ingredients and the used parts and percentages are presented in detail in Table 1. About 25% of the Loboob formulation that we used in our research contained 18 different medicinal herbal parts (four kernels, four seeds, three fruits, two roots, one oleo-gum-resin, one bark, one leaf, one bud, and one rhizome) that were added to honey and sugar to make the total of 20 components as shown in Table 1. Another 20-component formulation of Loboob with ten ingredients in common with the formulation that we used was described as effective for infertile men with sperm counts ≥0.5×10^6^/ml (Bioos *et al*., 2015).

Despite the improvement seen in mean sperm count, no dosage of Loboob showed statistically significant effect on mean sperm counts. According to the literature, several components of Loboob have been described to affect sperm count. *Cinnamomum zeylanicum Blume* (cinnamon) has a modulating role on heat stress and decreased testis apoptosis (Türk *et al*., 2015). *Sesamun indicum L* (sesame) might improve sperm count via its antioxidant properties (Khani *et al*., 2013). There is some controversy on the effects of *allium cepa* (onion) on sperm counts derived from the use of different parts of the plant (bulb *vs*. seeds). The antioxidant activity of the onion bulb and serum testosterone increases associated with onion seeds might be responsible for enhancements in sperm counts (Khaki *et al*., 2009; Vahdani & Khaki, 2014). Adeoye *et al*. (2018) reported possible negative dose dependent effects of the onion bulb on sperm counts. However, onion seeds were used to prepare Loboob in our study. The other contributing ingredient is *Alpinia officinarum Hance* (lesser galangal) with its component galangin leading to anti-oxidative effect (Kolangi *et al*., 2019). Effects arising from the anti-oxidant properties of *Withania somnifera* (ginseng) and hormonal effects caused by GABA mimetic features on gonadotropin releasing hormone, follicle-stimulating hormone, luteinizing hormone (LH) and testosterone have been described (Nasimi Doost Azgomi *et al*., 2018). *Zingiber officinale* (ginger) is supposed to affect sperm counts via its anti-oxidant properties, increases in testosterone and LH levels, and modification of nitric oxide levels leading to changes in vascular blood flow with final consequences on hormone production (Banihani, 2019). Incongruent to other ingredients, *Piper nigrum L.* (pepper) is supposed to have anti-spermatic effect (Mishra & Singh, 2009). Lack of an effect of Loboob on sperm count might be attributed to the powerful side effects of *Piper nigrum L* or other ingredients that have not been studied yet.

Loboob improved sperm motility in our study. All dosages of Loboob improved slow sperm while doses of 70 and 140 mg/kg had positive effects on progressive motility. *Cocos nucifera L.* (coconut) via its anti-oxidant properties in maintaining bioactivity of biological systems, cytokinins, and other components like sugar, vitamin, minerals and amino acids are considered effective at increasing sperm motility (Jimoh, 2020). Increasing blood serum omega-6 and plant source of omega-3 by *Juglans regia* (walnut)(Robbins *et al*., 2012), sugar component of *Corylus avellana L.* ( hazelnut) (Li & Ross, 1988), and anti-oxidant properties of *Sesamun indicum L* and *Withania somnifera* (Khani *et al*., 2013; Nasimi Doost Azgomi *et al*., 2018) might enhance motility. Although *allium cepa* may improve sperm motility by its antioxidant activity and alteration of ion channels permeability (Chae *et al*., 2017; Khaki *et al*., 2009), Adeoye *et al*. (2018) revealed a dose-dependent toxic effect of *allium cepa* bulb on sperm motility by increasing the incidence of abnormal sperm tail shapes. Our study used seeds of *allium cepa*, which might explain our different results. *Mentha piperita* L. (mint) may also improve motility (Tvrdá *et al*., 2018). *Prunus dulcis Mill.* (almond) enhances sperm motility via its unsaturated fatty acid component (Hussein & Raheem, 2015). Protecting structural and functional membrane integrity as well as mitochondrial effects are the mechanisms described for *Syzygium aromaticum* (cloves) as a dose-dependent component (Mishra & Singh, 2016; Santos *et al.*, 2019). Protecting structural and functional membrane integrity as well as mitochondrial effects are the mechanisms described for *Syzygium aromaticum* (cloves) as a dose-dependent component (36, 37). *Zingiber officinale* may also affect motility via magnesium supplementation and some enzymatic pathways (Banihani, 2019).

Treatment with 140 mg/kg Loboob improved sperm viability in our study. This result can be attributed to *Juglans regia* by its omega 3 ingredients (Robbins *et al*., 2012), antioxidant and inhibition of apoptosis via the action of poly unsaturated fatty acids of *allium cepa* (Khaki *et al*., 2009). *Prunus dulcis Mill*. is also supposed to enhance sperm viability (Hussein & Raheem, 2015). Sperm viability is enhanced by *Prunus dulcis Mill*. (35). Besides, *Syzygium aromaticum* positively affects sperm viability by protecting structural and functional membrane integrity (Santos *et al*., 2019). It should be considered that *Piper. cubeba* is used as a critical ingredient in many formulations such as Laboob-e-Sagheer (Bano *et al*., 2018). *Corylus colurna* is a component of various complex Unani formulations such as Khurma, Laboob-e- Sagheer, Laboob-e-Kabeer, and is used as an aphrodisiac when combined with honey (Akhtar *et al*., 2010).

Loboob did not statistically change the mean body weight of the rats included in the study. However, *Cinnamomum zeylanicum* Blume can increase body weight via its inhibitory effect on heat stress changes and by increasing appetite and feeding efficacy (Türk *et al*., 2015). *Corylus avellana L*. can affect via its lipid composition (Savage & McNeil, 1998). On the contrary, an anti-obesity role has been described for *Alpinia ofﬁcinarum Hance* in the literature due to the combined effects of various phytochemicals such as ﬂavonoids and phenolics (Xia *et al*., 2010). Loboob did not significantly increase the mean body weight of the rats with busulfan-induced subfertility included in this study.

Although improvements were seen in mean sperm morphology in the rats treated with Loboob, the differences in relation to untreated rats were not statistically significant. Micronutrients and omega 3 in *Juglans regia* (Robbins *et al*., 2012) are considered to improve sperm morphology. It should be noticed that there is some controversy about the impact of *allium cepa* on sperm morphology. Although Khaki *et al*. (2009) found that *allium cepa* did not affect sperm morphology, a toxic effect of *allium cepa* bulb on sperm morphology with findings such as absence of the head or tail and having an angulated or twisted tail was described in another study (Adeoye *et al*., 2018). High levels of reactive oxygen species (ROS) are detected among abnormally shaped sperms. *Alpinia officinarum Hance* might reduce the production of ROS via its phytochemicals, mainly galangin. It also diminishes apoptosis by caspase inhibition and regulation of Bcl‐2 and Bax expression (Kolangi *et al*., 2019). Another anti-oxidant that protects against DNA damage is *Prunus dulcis Mill* (Hussein & Raheem, 2015).

As far as we know, this is the first study that analyzed male rats with busulfan-induced subfertility to show and compare the protective effects of Loboob in different dosages on sperm parameters in a randomized trial. Our study showed that Loboob decreased some of the undesirable effects of busulfan on male rat sperm parameters in a dose-dependent manner. It is believed that this traditional multi-herbal formulation might induce similar valuable therapeutic effects not only after busulfan chemotherapy, but also for men in the general population suffering from infertility or subfertility as it has been traditionally used. We think that more studies with higher dosages of Loboob are required to find the best therapeutic dosage. We acknowledge the limitations of this study, which include the fact that it was performed on rats. Human studies using different Loboob dosages are warranted to see the possible effects or side effects of this herbal electuary as a possible human therapeutic modality. Different methods of preparation, extraction procedures, alterations in the bioavailability of herbal ingredients, and possible allergy or toxicity of the different components should be evaluated, especially in human studies. Future human studies may find a role for Loboob in the management of male subfertility or for protection of the testes from side effects of alkylating chemotherapeutic agents.

## CONCLUSION

This study showed that Loboob, a traditional Persian multi-herbal formulation used for male infertility, has dose-dependent protective effects on several sperm parameters in rats with busulfan-induced subfertility. Loboob improved the motility of slow spermatozoa in all three dosages used in this study, while progressive motility improved in dosages of 70 and 140mg/kg/day, and viability improved only in the dosage of 140mg/kg/day.
